# Inefficient integration between effort and reward in anhedonia

**DOI:** 10.1017/S0033291726103249

**Published:** 2026-03-03

**Authors:** Zhao Wang, Shiyu Zhou, Bo Gao, Haohan Sang, Ya Zheng

**Affiliations:** 1 Department of Mental Health and Psychology, Dalian Medical University, Dalian, China; 2 Department of Psychology, Guangzhou University, Guangzhou, China

**Keywords:** anhedonia, effort, motivation, neural dynamics, reward

## Abstract

**Background:**

Anhedonia is defined as a reduced interest in or inability to experience pleasure from reward-related activities. Recent studies have demonstrated deficient effort-based motivation in anhedonia, but the neural dynamics underlying the interface between effort and reward remain unclear.

**Methods:**

To address this issue, we recruited an anhedonia (ANH) group (*N* = 40) and a control (CNT) group (*N* = 40) to complete two tasks: (1) an effort–reward task where participants earned varying rewards by exerting different levels of physical effort and (2) an effort-based decision-making task where they chose between a no-effort option for a smaller reward and a high-effort option for a larger reward. We recorded EEG during both tasks and analyzed the resulting neural responses.

**Results:**

As expected, the ANH group showed reduced reward responses in both self-reported ratings and event-related potential (ERP) data in response to cue stimuli (indexed by the cue-P3) and reward feedback (indexed by the reward positivity). Importantly, the ANH group exhibited inefficient integration between effort and reward, showing an absent effort-discounting effect on the feedback-P3 during reward evaluation and a lack of reward-related theta modulation during effort-based decision-making.

**Conclusions:**

Our findings suggest a neurodynamic motivation model in anhedonia that informs precise interventions for relevant neuropsychiatric disorders.

## Introduction

Anhedonia, characterized by diminished interest or pleasure in rewarding activities, represents a core symptom of both depression and schizophrenia with negative symptom (Pelizza & Ferrari, 2009) and frequently manifests in neurological disorders such as Parkinson’s disease (Husain & Roiser, 2018). This condition correlates with severe social impairment and poor treatment outcomes (Craske et al., 2016). From a reward perspective, anhedonia involves multifaceted deficits of hedonic function, including interest, anticipation, motivation, cost–benefit computation, liking, and feedback integration (Pizzagalli, 2022). Critically, motivational impairment remains the least understood aspect of anhedonia (Treadway & Salamone, 2022).

Prior research demonstrates that individuals with anhedonia consistently exhibit diminished effort-based motivation, operationalized as reduced willingness to exert effort for rewards (Chong, Bonnelle, & Husain, 2016). Behavioral evidence from effort-based decision-making tasks confirms this deficit across subclinical (Slaney et al., 2023; Wang et al., 2018) and clinical (Barch, Treadway, & Schoen, 2014; Yang et al., 2014) populations. Functional magnetic resonance imaging (fMRI) studies further reveal anhedonia-related dysregulation in core reward circuits (the anterior cingulate cortex, prefrontal cortex, and striatum) during effort–reward tradeoff (Bi et al., 2022; Huang et al., 2016; Wang et al., 2022). Crucially, while prior research has centered on effort-based decision-making, reward motivation is a dynamic, multifaceted process (Husain & Roiser, 2018). Thus, abnormal effort-based motivation in anhedonia likely extends beyond decision-making to impair other reward aspects including anticipatory and consummatory experiences.

Event-related potentials (ERPs) provide millisecond-level temporal resolution, enabling precise tracking of the neural dynamics underlying the interaction between reward processing and effort expenditure, which cannot be achieved with slower neuroimaging methods such as fMRI due to its sluggish BOLD response. During the anticipatory phase, the cue-P3 is more positive for reward versus neutral cues and for high- versus low-reward cues (Broyd et al., 2012; Zhang et al., 2017), reflecting categorizing a cue to prepare for upcoming events. Critically, the cue-P3 shows decreased amplitudes for high- versus low-effort cues (Zheng, Zhang, & Wu, 2024) and predicts subsequent effort investment (Frömer et al., 2021). During the consummatory phase, reward positivity (RewP) serves a neural signature for reward sensitivity (Proudfit, 2015) or reward prediction error (Holroyd & Coles, 2002), while the feedback-P3 reflects motivational salience toward outcomes (Nieuwenhuis, Aston-Jones, & Cohen, 2005). Effort expenditure dynamically modulated both the RewP and feedback-P3, with directionality contingent upon effort type, perceived control, and reward beneficiary (Bogdanov et al., 2022; Bowyer et al., 2021; Jiang & Zheng, 2023; Wu & Zheng, 2023; Zheng & Tang, 2024). Additionally, the successful completion of effortful tasks elicited an enhanced performance-P3 (Bowyer et al., 2021), signaling intrinsic motivation derived from effort performance.

Previous ERP research reveals consistent abnormalities in anticipatory reward processing in anhedonia, characterized by a diminished cue-P3 response to monetary reward cues (Chen, Xu, Zhou, & Zheng, 2018; Santopetro et al., 2021; Zhou et al., 2019). Findings during consummatory reward processing, however, are less consistent. Whereas some studies reported reduced RewP and feedback-P3 amplitudes in anhedonia (Chen et al., 2018; Liu et al., 2013; Morie, De Sanctis, Garavan, & Foxe, 2016; Padrão et al., 2013; Parvaz, Gabbay, Malaker, & Goldstein, 2016; Santopetro et al., 2021; Wang, Li, Nie, & Zheng, 2020; Zhou et al., 2019), others found no abnormalities (Barch et al., 2020; Frank, Stevens, & Versace, 2019; Mueller et al., 2015). A critical factor explaining this discrepancy appears to be task effort requirement. Studies reporting null findings typically employed low-effort tasks (e.g. gambling or probability learning; Barch et al., 2020; Frank et al., 2019; Mueller et al., 2015), whereas those observing ERP deficits often utilized effort-demanding paradigms like the money incentive delay task (Morie et al., 2016; Zhou et al., 2019). This pattern highlights effort as a key modulator in the relationship between reward processing and anhedonia (Husain & Roiser, 2018). Supporting this view, Wen, Wu, Wang, Gao, and Zheng (2024) demonstrated using an effort-based monetary incentive delay task that anhedonia was characterized by reward-insensitive effort enhancement of the feedback-P3, that is, effort amplified neural responses irrespective of rewards availability. While this highlights that reward and effort jointly contribute to abnormal neural signals in anhedonia, the complete neural dynamics of this interplay remain poorly understood.

In this study, we addressed this issue by asking a high anhedonia (ANH) group and a healthy control (CNT) group to complete an effort–reward task in which they earned varying rewards by exerting different levels of physical effort. Neural responses were recorded during both the anticipatory (indexed by the cue-P3) and consummatory (indexed by the performance-P3, RewP, and feedback-P3) phases. Based on the established reward processing deficits in anhedonia, we hypothesized that the ANH group would show reduced reward effects across the cue-P3, RewP, and feeback-P3 components. Importantly, these reward effects would be discounted more strongly in the ANH group relative to the CNT group, reflecting their diminished willingness to invest effort for rewards (Slaney et al., 2023; Wang et al., 2018). We additionally explored group differences in the performance-P3 following successful effort exertion. However, no specific hypothesis was made due to limited prior evidence.

We also examined EEG responses underlying effort-based decision-making. The two groups further completed an effort-based decision-making task in which they chose between a no-effort/small-reward option and a high-effort/large-reward option. We focused on frontal midline theta (4–7 Hz) response to decision options due to its established association with decision conflict (Cavanagh & Frank, 2014; Umemoto, Lin, & Holroyd, 2023). While prior research shows altered theta activity in anhedonia during reward processing (Mueller et al., 2015; Padrão et al., 2013), effort-related theta modulation remains unexplored in this population. Given theta’s role in cost–benefit integration and anhedonia’s motivational deficits, we hypothesized that the ANH group would show enhanced theta activity relative to the CNT group during decisions requiring the integration of reward value and effort costs.

## Material and methods

### Participants

An ANH group (*N* = 40) and a CNT group (*N* = 40) were recruited from a larger sample of 1510 university students (357 males, 1153 females) based on their scores on the Chinese version of the Revised Chapman Physical Anhedonia Scale, which comprises 61 true–false items assessing individual differences in the capacity to experience pleasure from physical and sensory stimuli (Chan et al., 2012; Chapman, Chapman, & Raulin, 1976; α = 0.86 in this sample). Based on previous studies (Wen et al., 2024), participants were assigned to the groups according to their scores relative to the sample distribution (*M* = 13.97, standard deviation [*SD*] = 8.05). The ANH group comprised individuals scoring >1.0 *SD* above the mean, while the CNT group comprised those >1.0 *SD* below the mean (Table 1). All participants were right-handed, had normal or corrected-to-normal vision, and were free of current psychiatric or neurological disorders. This was confirmed through the administration of the Structured Clinical Interview for DSM-5 (Research Version) by two professionally trained research staff members. Participants provided written informed consent and received monetary compensation, including task earnings. This study was approved by the Dalian Medical University Institutional Review Board.

**Table 1.** Demographic characteristics and behavioral data (*M* ± *SD*)

	CNT(*N* = 40)	ANH(*N* = 40)	^t^/χ^2^	*p*	Cohen’d
Gender (M/F)	12/28	10/30	0.25	.617	–
Age (years)	19.80 ± 1.86	19.50 ± 1.54	0.79	.433	0.18
Education (years)	13.63 ± 1.82	13.30 ± 1.42	0.89	.376	0.20
RPAS	4.47 ± 1.71	29.68 ± 5.82	−26.28	< .001	−5.88
BDI-II	3.98 ± 5.46	10.30 ± 9.11	−3.77	< .001	−0.84
TEPS	93.55 ± 9.03	73.30 ± 11.86	8.59	< .001	1.92
Anticipatory pleasure	42.83 ± 4.48	33.25 ± 6.85	7.40	< .001	1.66
Consummatory pleasure	50.73 ± 5.77	40.05 ± 7.21	7.31	< .001	1.64
BAS					
Drive	12.50 ± 1.81	11.10 ± 2.79	2.66	.010	0.60
Fun seeking	15.50 ± 1.65	14.08 ± 2.47	3.03	.003	0.68
Reward responsiveness	13.70 ± 1.31	12.75 ± 1.78	2.72	.008	0.61
BIS	14.98 ± 2.22	15.10 ± 2.60	−0.23	.818	−0.05
MBP	32.44 ± 4.85	32.62 ± 4.87	−0.16	.873	−0.04
Success rates (%)	97.77 ± 1.84	97.26 ± 2.46	1.05	.298	0.23

Abbreviations: CNT = the control group; ANH = the anhedonia group; RPAS = Revised Physical Anhedonia Scale; BDI-II = Beck Depression Inventory II; TEPS = Temporal Experience of Pleasure Scale; BAS = Behavioral Activation System; BIS = Behavioral Inhibition System; MBP = Maximum Button Press.

### Procedure

Participants sequentially performed an effort–reward task and an effort-based decision-making task while their EEG was recorded. Following these tasks, participants completed a 9-point Likert scale (1 = not at all, 9 = very much) to rate their experiences across different effort levels, including liking, required effort, fatigue, time pressure, and performance. They also completed the Temporal Experience of Pleasure Scale (TEPS; Gard, Gard, Kring, & John, 2006), the Beck Depression Inventory II (BDI-II; Beck, Steer, & Brown, 1996), and the Behavioral Inhibition System/Behavioral Activation System Scales (BIS/BAS; Carver & White, 1994).

**Figure 1.**
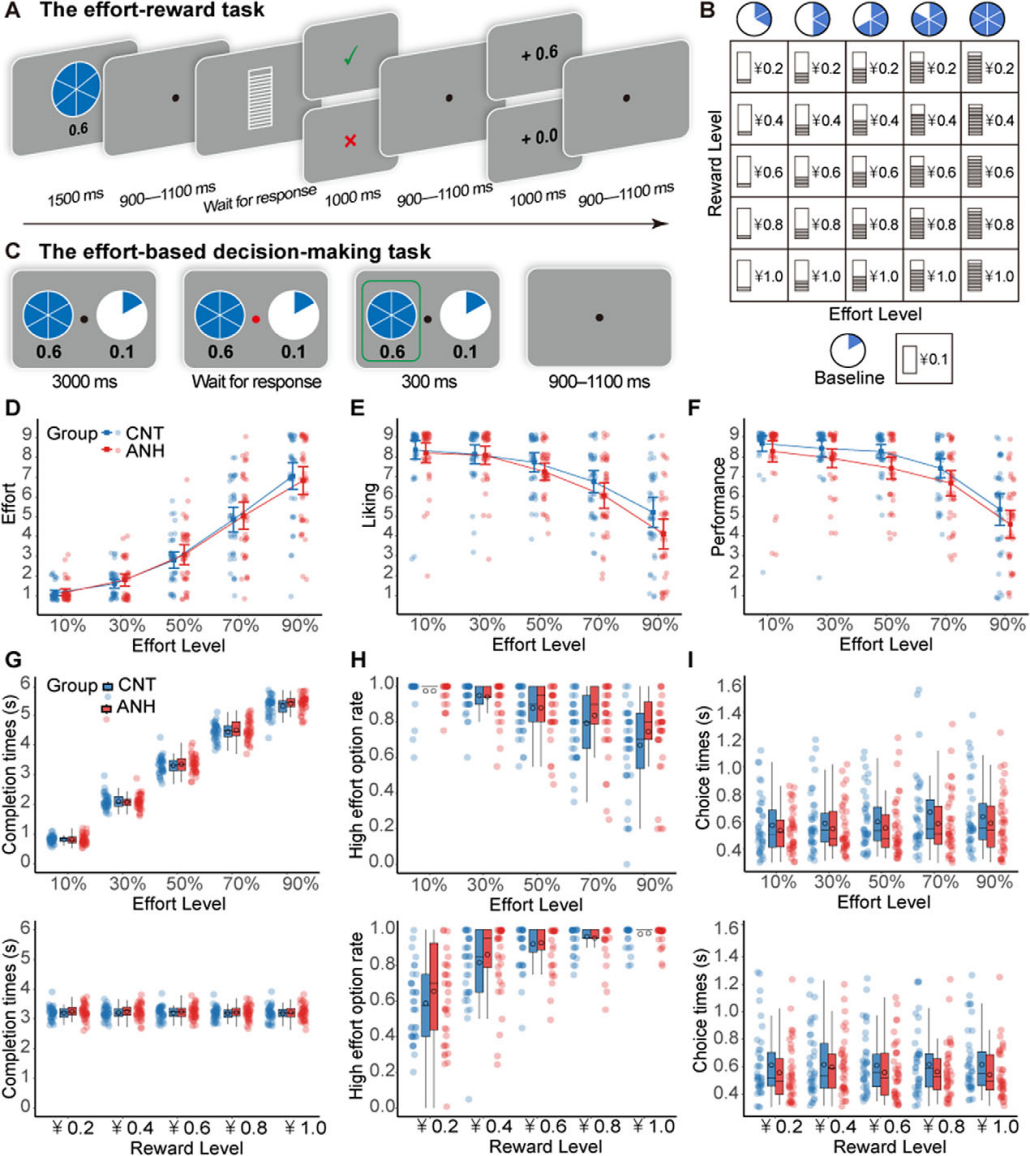
Experimental tasks, rating, and behavioral results. (A) The effort–reward task. Participants exerted physical effort at varying levels (10%, 30%, 50%, 70%, or 90% of their MBP) to earn rewards ranging from ¥0.2 to ¥1.0 in ¥0.2 increments. Successful effort exertion resulted in a 50% chance of winning the reward. (B) The offered reward and effort levels in the effort–reward task and the effort-based decision-making task. The two variables were orthogonal in the both tasks. (C)The effort-based decision-making task. Participants chose between a baseline no-effort option for a smaller reward and a high-effort option for a larger reward. Rating and behavioral results. (D–F) Rating data of perceived effort (D), liking (E), and performance (F). (G–I) Behavioral data of completion times for the effort–reward task (G), high-effort acceptance rates (H), and choice times (I) for the effort-based decision-making task. In the effort–reward task, completion time increased with effort and decreased with reward. Crucially, performance was comparable across the CNT and ANH groups. In the decision-making task, the ANH group appeared less sensitive to increasing effort costs than the CNT group when deciding whether to exert effort. Error bars represent the within-subject standard error of the mean.

**The effort**–**reward task.** This task was designed to measure neural responses to rewards obtained after physical effort exertion (Figure 1A). Before the main task, participants completed three 6000-ms trials where they pressed a button as fast as possible using their non-dominant pinky finger. The maximum button press (MBP) was calculated as the average press number across these trials (Zheng & Tang, 2024). The MBP was 32.44 ± 4.85 for the CNT group and 32.62 ± 4.87 for the ANH group (*p* = .873). In the main task, each trial began with a 1500-ms cue displaying two pieces of information: a pie chart showing the required effort level (10%, 30%, 50%, 70%, or 90% of participants’ MBP) and a number indicating the potential reward (¥0.2, ¥0.4, ¥0.6, ¥0.8, or ¥1.0). The five effort levels were fully crossed with the five rewards, yielding 25 unique combinations (Figure 1B). Following a jittered interval (900–1100 ms), participants entered the effort-execution phase. They had 6000 ms to complete the required button presses with their non-dominant pinky finger. A line in a rectangular box provided real-time feedback by moving upward with each press. The phase terminated upon reaching the target line. After another jittered interval (900–1100 ms), a performance feedback stimulus was shown for 1000 ms, indicating whether the required effort level was achieved. If successful, a tick appeared for 1000 ms, signaling eligibility for the cued reward. Following another jittered interval (900–1100 ms), a utilitarian feedback stimulus was displayed for 1000 ms, indicating whether the reward was received or not. Gains and nongains were equally likely and delivered pseudorandomly. Each trial ended with a jittered interval (900–1100 ms). If participants failed to achieve the required effort level, a cross appeared, and the trial ended after a jittered interval (900–1100 ms). The task consisted of 150 trials divided into six blocks of 25 trials. Participants completed six practice trials before the main task.

**The effort-based decision-making task.** This task was designed to measure participants’ willingness to exert physical effort for rewards (Figure 1C). Each trial began with a baseline no-effort option for ¥0.1 and a high-effort option for a larger reward. The high-effort option had 25 unique effort–reward combinations identical to those in the effort–reward task. The options were presented for 3000 ms, after which the central fixation point turned red. To minimize motor response confounds, participants waited for this color change before indicating their choice within 5000 ms by pressing the ‘F’ or ‘J’ key with their left or right index finger. The selected option was highlighted with a green box for 300 ms. Each trial ended with a jittered interval (900–1100 ms). The task consisted of 100 trials divided into four blocks. To maintain participant focus, we included 20 catch trials requiring participants to verify the effort and reward levels of the high-effort option. Seven participants (four from the CNT group and three from the ANH group) had an accuracy rate below 60% on these trials. Nonetheless, excluding these participants led to no reliable differences in result pattern. To ensure incentive compatibility, participants were informed that some trials would be randomly selected to determine their final payment, but they were not told exactly how many trials would be selected. They were encouraged to consider each decision carefully since each trial had an equal chance of being selected. Participants completed five practice trials before the task and completed their chosen options for the 20 selected trials post-task.

### EEG recording and processing

EEG data were recorded during both tasks. For the effort–reward task, we measured trial-level ERP components, including (1) the cue-P3 (440–600 ms post-cue onset), (2) the performance-P3 (250–450 ms post-performance feedback), and (3) both RewP (276–376 ms) and feedback-P3 (324–424 ms) following reward feedback. Additionally, trial-level theta power (100–400 ms over 4–7 Hz) during option evaluation was measured in the effort-based decision-making task. Complete preprocessing and analysis pipelines are detailed in the Supplemental Materials.

### Data analysis

Our key statistical analyses were based on mixed-effects regression models with random intercepts and slopes (unstructured covariance matrix), implemented in the lme4 package v1.1.31 (Bates, Mächler, Bolker, & Walker, 2015) in R v4.4.0. For the effort–reward task, we analyzed behavioral data (success rates and completion times) and ERP data of cue-P3 and performance-P3 using a linear mixed-effects regression model, with group, effort, reward, and their interactions as predictors. RewP and feedback-P3 data were fitted by including an additional predictor of valence. For the effort-based decision-making task, we fitted choice data using a mixed-effects logistic regression model with a binomial link function, and data of decision time and theta power using a linear mixed-effects regression model, with group, effort, reward, and their interactions as predictors. For all models, we contrast-coded categorical regressors (group: −0.5 for CNT and + 0.5 for ANH; valence: −0.5 for nogain and + 0.5 for gain) and z-scored continuous regressors (effort and reward) within participants. We determined random effects for each model using singular value decomposition to report the maximal possible random effect structure. We excluded trials with failed responses (2.22% for the CNT group and 2.73% for the ANH group) in the effort–reward task and trials with no responses (0.13% for the CNT group and 0.08% for the ANH group) in the effort-based decision-making task from statistical analyses.

## Results

### Results of demographic and rating data

#### Anhedonia is characterized by weaker pleasure experience and higher depression level

The ANH group showed significantly higher depression levels on the BDI-II scores compared to the CNT group, along with lower anticipatory and consummatory pleasures on the TEPS. The ANH group also exhibited significantly lower scores on drive, fun seeking, and reward responsiveness in the behavioral activation system, while the scores of the behavioral inhibition system were comparable between the two groups (Table 1).

#### Anhedonia is associated with increased effort disliking and poorer self-rated performance

Participants perceived increased effort as more effortful (*b* = 2.09, *p* < .001), more fatiguing (*b* = 2.08, *p* < .001), and more time-pressured (*b* = 2.05, *p* < .001). They also found it less likable (*b* = −1.27, *p* < .001) and were less satisfied with their performance (*b* = −1.15, *p* < .001). Compared to the CNT group, the ANH group showed comparable effort perception (Figure 1D) but experienced a steeper decline in liking ratings as required effort increased (*b* = −0.36, *p* = .008; Figure 1E). Although the ANH group performed similarly to the CNT group in the effort–reward task, they rated their performance more negatively (*b* = −0.65, *p* = .019; Figure 1F). No other significant effects were found (Supplementary Table S1).

### Results of the effort–reward task

**Figure 2.**
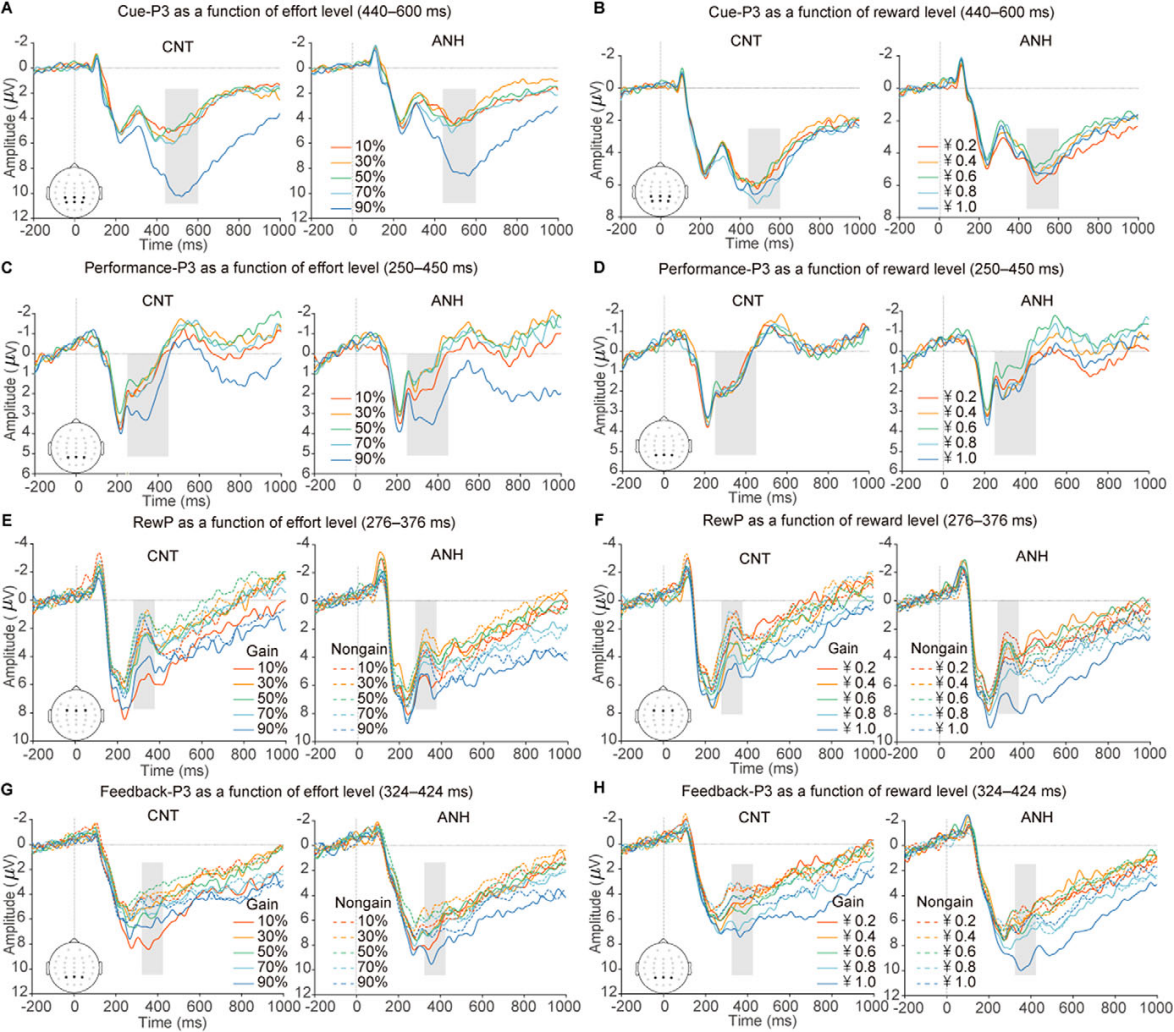
Grand-averaged ERP waveforms elicited during the effort–reward task, showing responses over centroparietal and parietal areas (A–B) during the cue-evaluation stage, over parietal areas (C–D) during the performance-evaluation stage, and over frontocentral (E–F) and parietal (G–F) areas during the reward-evaluation stage. The ERP waveforms are depicted as a function of effort (left) and reward (right) levels, separately for the CNT group and the ANH group. Shaded vertical bars show the time windows for quantification.

Behavioral data for the effort–reward task are shown Figure 1G (see detailed statistical results in Supplemental Materials). Figure 2 depicts grand-averaged ERP waveforms in response to cue stimuli, performance feedback, and reward feedback. Topographic maps of these ERP components are shown in Supplementary Figure S1.

#### Anhedonia is linked to an absent reward-enhancement effect during the cue-evaluation stage

**Figure 3.**
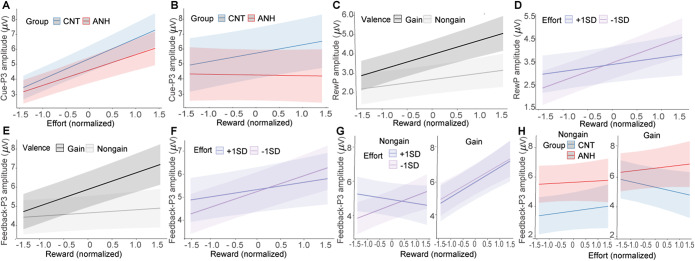
ERP results of the effort–reward task. (A–B) Fixed effects of effort (A) and reward (B) on the cue-P3 as a function of group. (C–D) Fixed effects of reward on the RewP as a function of valence (C) and effort (D). (E–G) Fixed effects of reward on the feedback-P3 as a function of valence (E), effort (F), and the interaction between valence and effort (G). (H) Fixed effects of effort on the feedback-P3 as a function of the interaction between valence and group.

During the cue-evaluation stage, the cue-P3 was increased with required effort (*b* = 1.20, *p* < .001; Figure 3A). Moreover, the cue-P3 tracked a significant interaction between group and reward (*b* = −0.31, *p* = .037; Figure 3B). Follow-up simple slopes analyses revealed that while the CNT group exhibited increased cue-P3 as potential reward increased (*b* = 0.29, *p* = .007), the ANH group showed no reward-related enhancement (*b* = −0.02, *p* = .818).

#### Anhedonia is associated with no effort-discounting effect on the feedback-P3 in response to gains during the feedback-evaluation stage

The RewP increased with reward level (*b* = 0.54, *p* < .001) and was larger for gains versus nongains (*b* = 1.26, *p* < .001). The reward effect was more pronounced for gains (*b* = 0.75, *p* < .001) than for nongains (*b* = 0.33, *p* = .015), as demonstrated by a significant interaction between reward and valence (*b* = 0.42, *p* = .014; Figure 3C). Also, the reward effect was decreased when invested effort was high (+1 *SD*; *b* = 0.30, *p* = .027) than when it was low (−1 *SD*; *b* = 0.78, *p* < .001), evidenced by a significant interaction between reward and effort (*b* = −0.24, *p* = .005; Figure 3D). Moreover, the ANH group exhibited a greater RewP than the CNT group (*b* = 1.57, *p* = .025), along with a significant interaction between group and valence (*b* = −0.85, *p* = .041). Post-hoc comparisons showed that the valence effect was less pronounced in the ANH group (*b* = −0.83, *p* = .006) than in the CNT group (*b* = −1.68, *p* < .001). No other significant effects were found (Supplementary Table S2).

The feedback-P3 increased with reward amount (*b* = 0.52, *p* < .001). It tracked significant interactions between reward and valence (*b* = 0.71, *p* < .001; Figure 3E) and between reward and effort (*b* = −0.19, *p* = .022; Figure 3F). These interactions were further modified by a significant three-way interaction among effort, reward, and valence (*b* = 0.42, *p* = .012). Follow-up simple slopes analyses (Figure 3G) revealed that the reward effect for gains was comparable regardless of invested effort being high (+1 *SD*; *b* = 0.89, *p* < .001) or low (−1 *SD*; *b* = 0.85, *p* < .001). For nongains, the reward effect was present when invested effort was low (−1 *SD*; *b* = 0.56, *p* = .001) but not when it was high (+1 *SD*; *b* = −0.23, *p* = .189). Furthermore, we found a significant three-way interaction among group, effort, and valence (*b* = 0.70, *p* = .033; Figure 3H). Follow-up simple slopes analyses showed that for gains, the feedback-P3 significantly decreased as invested effort increased in the CNT group (*b* = −0.37, *p* = .025) but not in the ANH group (*b* = 0.21, *p* = .215). For nongains, the feedback-P3 did not vary with effort level for both the CNT group (*b* = 0.23, *p* = .163) and the ANH group (*b* = 0.10, *p* = .533).

Finally, we found a main effort effect on the performance-P3 in response to success feedback (i.e. effort-completion cues), with its amplitudes increasing as a function of invested effort (*b* = 0.49, *p* < .001). Importantly, the effort-enhancement effect was comparable between the ANH and CNT groups, as revealed by the absence of the group-by-effort interaction (*b* = 0.07, *p* = .756). No other significant effects were found (Supplementary Table S2).

### Results of the effort-based decision-making task

**Figure 4.**
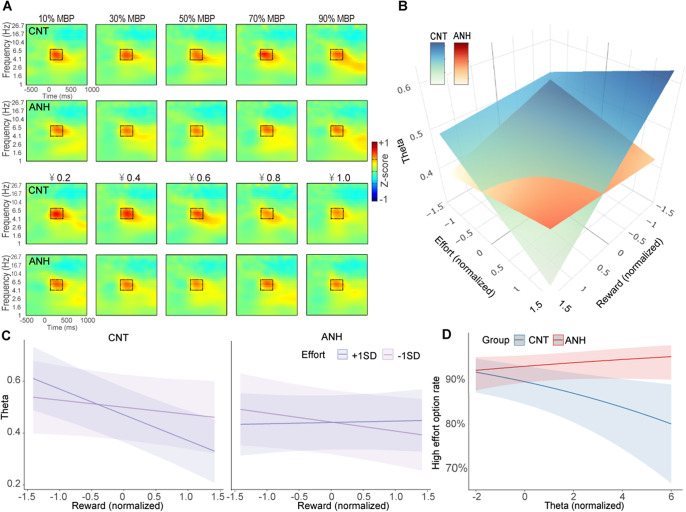
Theta results in the effort-based decision-making task. (A) Time–frequency representations of EEG power at FCz. The black boxes depict time–frequency windows (100 to 400 ms over 4–7 Hz) for quantification. (B–C) Fixed effects of effort and reward on theta power as a function of group, with effort displayed either continuously (B) or categorically (C). (D) Fixed effects of theta power on high-effort acceptance rates as a function of group.

Behavioral data from the effort-based decision-making task are shown Figure 1H,I (see detailed statistical results in Supplemental Materials). Figure 4A depicts time–frequency representations of EEG power in response to decision options.

#### Anhedonia is linked to abnormal neural tradeoff between reward and effort

One participant from the CNT group was excluded from theta power results due to insufficient artifact-free EEG trials (less than 50% of total trials). Theta power elicited by decision options was decreased as reward level increased (*b* = −0.04, *p* < .001). Importantly, we found a significant interaction between reward and group (*b* = 0.05, *p* = .028), which was further qualified by a significant three-way interaction among group, reward, and effort (*b* = 0.06, *p* = .012; Figure 4A–C). Post-hoc simple slopes analyses revealed that when required effort was high (+1 *SD*), theta power decreased as reward level increased in the CNT group (*b* = −0.10, *p* < .001) but not in the ANH group (*b* = 0.01, *p* = .814). When required effort was low (−1 *SD*), theta power did not vary with reward level across the CNT (*b* = −0.03, *p* = .219) and ANH groups (*b* = −0.03, *p* = .115). No other significant effects were found (Supplementary Table S2). These results indicate that anhedonic individuals could not trigger cognitive control appropriately when deciding to invest effort to obtain rewards.

Next, we examined whether theta power could predict participants’ decision-making behavior using a mixed-effects logistic regression model with a binomial link function. The model predictors included group, theta power (z-scored within participants), and their interaction. We found a significant interaction between group and theta power (*b* = 0.19, *p* = .006). Follow-up simple slopes analyses (Figure 4D) showed that theta power negatively predicted high-effort choices in the CNT group (*b* = −0.12, *p* = .006) such that enhanced theta power was associated with decreased willingness to choose the high-effort option. However, this effect was not observed in the ANH group (*b* = 0.07, *p* = .220). These results suggest that anhedonic individuals may have difficulty in balancing reward and effort to guide their decision-making.

## Discussion

This study investigated the neural dynamics underlying reward–effort integration in anhedonia. We recorded EEGs from the ANH and CNT groups during both an effort–reward task and an effort-based decision-making. As expected, the ANH group exhibited blunted reward responsiveness, evidenced by reduced self-reported ratings and attenuated neural responses to both reward-predicting cues (the cue-P3) and reward feedback (the RewP). Importantly, the ANH group demonstrated inefficient integration between reward and effort, characterized by an absent effort-discounting effect on the feedback-P3 during reward evaluation and a lack of reward-related theta modulation during effort-based decision-making.

### Reduced extrinsic motivation but intact intrinsic motivation in anhedonia

Our study replicates prior findings of reduced reward sensitivity in anhedonia. Self-reported data revealed significantly lower scores in the ANH compared to CNT group across both anticipatory and consummatory pleasures of the TEPS and all BAS subscales (drive, fun seeking, and reward responsiveness). ERP results demonstrated that anhedonia was associated with diminished reward sensitivity in the effort–reward task. During reward anticipation, the CNT group exhibited enhanced cue-P3 amplitudes as potential reward increased, reflecting heightened motivational salience for reward prospect (Frömer et al., 2021; Zhang et al., 2017). However, the ANH group exhibited absent cue-P3 reward modulation. During feedback evaluation, the ANH group showed attenuated RewP differentiation between gains and nongains compared to the CNT group (Liu et al., 2013; Parvaz et al., 2016). These convergent self-reported and ERP findings demonstrate impaired reward dynamics from anticipation to consumption in anhedonia (Kieslich, Valton, & Roiser, 2022; Pizzagalli, 2022; Romer Thomsen, Whybrow, & Kringelbach, 2015). This impairment constitutes a core mechanism underlying motivational deficits that explains diminished reward-seeking behaviors and reduced engagement in pleasurable activities among anhedonic individuals.

Unlike deficits in monetary reward motivation, our findings indicate preserved effort processing in anhedonia. Behaviorally, the ANH group showed similar performance compared to the CNT group during the effort–reward task, with no group differences in MBP, success rates, or task completion times. Neurally, both groups showed similar effort–enhancement effects on the cue-P3 during effort anticipation. These results are consistent with recent evidence of intact motor preparation in anhedonia (Wen et al., 2024). Furthermore, the ANH group, like the CNT group, showed an increased performance-P3 when viewing feedback that higher required effort was achieved. Given that the P3 reflects motivational salience toward feedback evaluation (Nieuwenhuis et al., 2005), this suggests that anhedonic individuals could derive value from effortful achievement (Bowyer et al., 2021; Jiang & Zheng, 2023). The effort-related P3 modulation may reflect intact intrinsic motivation (e.g. pride, achievement) following successful performance in anhedonia. Together, these observations indicate preserved physical effort processing and its associated intrinsic motivation in anhedonia.

### Inefficient integration between physical effort and reward evaluation in anhedonia

The novel finding of this study is the inefficient integration between physical effort exertion and reward evaluation in anhedonia. Specifically, the CNT group exhibited an effort-discounting effect on gain-elicited feedback-P3 with its amplitude being significantly decreased as invested effort increased (Westbrook, Kester, & Braver, 2013). This reflects subjective utility of rewards following high effort due to increased exertion costs (Bowyer et al., 2021). Crucially, the ANH group exhibited no such effort-discounting of feedback-P3 responses to gains. This indicates that anhedonic individuals have difficulties in integrating effort costs with reward benefits, failing to use computations that devalue rewards proportionally to expended effort (Westbrook & Braver, 2015). This dysfunctional effort–reward valuation is consistent with recent evidence of amplified feedback-P3 valence effects after high cognitive effort in anhedonia regardless of reward availability (Wen et al., 2024). Collectively, these results demonstrate an inefficient cost–benefit analysis in anhedonia. Anhedonic participants appear unable to accurately integrate effort costs into reward evaluation, resulting in the absent effort-discounting effect. This failure to devalue rewards in proportion to effort expended signifies a fundamental impairment in adaptive resource allocation. Presumably, such blunted effort-discounting would lead to a systematic overestimation of the utility of demanding activities, driving maladaptive choices where considerable energy is invested for minimal returns. This pattern may contribute to the fatigue, inefficiency, and sense of pointlessness often observed in anhedonia (Husain & Roiser, 2018).

The inefficient reward–effort integration in anhedonia was further supported by theta findings from the effort-based decision-making task. Originating from the anterior cingulate cortex (Cavanagh & Cohen, 2022), frontal midline theta is thought to reflect cognitive conflict and decision difficulty (Cavanagh & Frank, 2014; Umemoto et al., 2023). In our effort-based decision-making task, participants chose between a baseline no-effort/small-reward option and a high-effort/large-reward option. In trials requiring high effort, the CNT group showed decreasing theta power with increasing rewards, indicating adaptive conflict reduction through reward valuation. In contrast, the ANH group exhibited no such reward buffering effect on effort-related theta activity, suggesting impaired conflict resolution through reward benefits. Furthermore, we found that theta power negatively predicted participants’ high-effort choices, reflecting that greater decision conflict decreases the willingness to invest high effort for larger rewards. However, this relationship between theta power and effort choices was observed for the CNT group but not for the ANH group. This finding aligns with prior observations of abnormal reward-related theta in anhedonia (Padrão et al., 2013) but goes beyond it to demonstrate dysregulated conflict signaling specifically during effort–reward computation. Together with the absent effort-discounting effect on the feedback-P3, the theta findings confirm the inefficient cost–benefit computation in anhedonia. Given the anterior cingulate cortex’s role in generating theta oscillations (Cavanagh & Cohen, 2022), computing effort–reward tradeoffs (Arulpragasam, Cooper, Nuutinen, & Treadway, 2018; Klein-Flügge, Kennerley, Friston, & Bestmann, 2016), and optimizing reward-seeking decisions (Brassard et al., 2024), our data implicate dysregulation in this area as the neural substrate underlying impaired reward–effort integration in anhedonia.

### A neurodynamic motivation model in anhedonia

Integrating the findings from both tasks, we propose a neurodynamic motivation model for anhedonia. Despite a lack of complete agreement, there are several temporally proximal processes that underlie amotivation in anhedonia, including option evaluation, effort–reward decision, effort exertion, effort completion, and effort–reward integration (Husain & Roiser, 2018). Leveraging EEG’s millisecond resolution, this study provides the first characterization of the neural dynamics underlying the effort–reward integration in anhedonia. We found that anhedonia shows reduced extrinsic motivation related to monetary rewards, ranging from reward anticipation (indexed by the cue-P3) to consumption (indexed by the RewP). While anhedonia preserves intrinsic motivation related to effort achievement (indexed by the performance-P3), it is best characterized by an inefficient interaction between effort and reward during decision-making (indexed by theta oscillation) and outcome evaluation (indexed by the fb-P3). This model requires validation across other effort domains (e.g. cognitive effort) and reward types (e.g. social reward) to establish generalizability. Given that anhedonia is transdiagnostic and serves as a core element in the framework of the Research Domain Criteria (Insel et al., 2010), this neurodynamic motivation model informs precise interventions for relevant neuropsychiatric disorders.

### Limitations and future directions

Several limitations should be acknowledged. First, participants took longer to complete higher effort trials, introducing confounding influences of time costs. However, this influence may be minimal given the relatively small completion time differences between the lowest and highest effort levels (Weinberg, Luhmann, Bress, & Hajcak, 2012). Second, effort information was more perceptually salient than reward information during cue evaluation, possibly attenuating reward-related neural effects. Third, examining effort-based reward dynamics and decision-making in separate tasks – while optimizing trial numbers and signal-to-noise ratios – risks introducing cross-task interference. Future studies should integrate these processes within a unified paradigm. Forth, our sample consisted of young, healthy subclinical individuals. Future studies should replicate our findings with clinical populations with anhedonia (e.g. major depressive disorder, schizophrenia with negative symptoms) to establish translational relevance.

## Conclusion

By combining an effort–reward task with an effort-based decision-making task, we demonstrate that anhedonia involves not only deficient reward processing but also inefficient integration between reward and effort. The impairment is characterized by the lack of effort-discounting during reward evaluation and the absence of reward modulation of cognitive conflict during effort-based decision-making. Our findings establish inefficient reward–effort as a core mechanism underlying motivational deficits in anhedonia.

## Supporting information

10.1017/S0033291726103249.sm001 Wang et al. supplementary material Wang et al. supplementary material

## Data Availability

Data and code that support the findings of this study are available on Open Science Framework at https://osf.io/6wzk5/?view_only=d33469d402734b6f89d638f115dd6405.

## Supplementary material

The supplementary material for this article can be found at http://doi.org/10.1017/S0033291726103249.
