# Supplementary material for: Inefficient integration between effort and reward in anhedonia
Source: Psychol Med. 2026 Mar 3;56:e62. doi: 10.1017/S0033291726103249 (PMC12969199; doi:10.1017/S0033291726103249)
Supplement: Wang et al. supplementary material [file S0033291726103249sup001.docx]

**Supplementary Materials**

**1 Supplementary Methods**

***1.1 EEG recording and processing***

EEG data were recorded using 28 Ag/AgCl channels placed on an elastic cap according to the international 10–20 system. Two additional channels were positioned on the left and right mastoids. The EEG were recorded with a reference channel placed between Cz and CPz. Horizontal and vertical electrooculograms (EOG) data were recorded from two pairs of channels over the external canthi of each eye and the left suborbital and supraorbital ridges, respectively. Both EEG and EOG signals were amplified using a Neuroscan Grael 4K amplifier with a low-pass filter of 100 Hz in DC acquisition mode and digitized at a rate of 512 samples per second. Channel impedances were maintained below 5 KΩ.

EEG data were analyzed using EEGLAB v2021.0 ([Delorme & Makeig, 2004](#_ENREF_19)) and ERPLAB v8.10 ([Lopez-Calderon & Luck, 2014](#_ENREF_31)) toolboxes in MATLAB 2020b (MathWorks, US). The signals were rereferenced to the average of the left and right mastoids and filtered with a bandpass of 0.1–35 Hz using a zero phase-shift Butterworth filter (12 dB/octave roll-off). Channels with poor quality or excessive noise were interpolated using the spherical interpolation algorithm, and portions of EEG containing extreme voltage offsets or break periods were removed. Ocular artifacts were removed using an infomax independent component analysis on continuous EEG data. Epochs were then extracted from -200 to 1000 ms relative to stimulus (choice or feedback) onset, with the prestimulus average activity as the baseline. An automatic artifact detection algorithm was applied to remove epochs with a voltage difference exceeding 50 μV between sample points or 200 μV within a trial, a maximum voltage difference less than 0.5 μV within 100-ms intervals, or a slow voltage drift with a slope greater than ±100 μV. On average, 96.96% of trials were retained for the effort-reward task and 95.64% of trials for the effort-based decision-making task.

ERP components elicited in the effort-reward task were measured at the trial level as the mean activity during different time windows averaged across channels over regions of interest. Measurement parameters were determined using a collapsed localizer approach ([Luck & Gaspelin, 2017](#_ENREF_32)) based on visual inspection of grand-averaged ERP waveforms and topographic maps collapsed across trials and participants. Specifically, the cue-P3 was scored as the mean voltage from 440 to 600 ms post cue onset over centroparietal areas (CP3/CPz/CP4/P3/Pz/P4), the performance-P3 from 250 to 450 ms post performance feedback onset over parietal areas (P3/Pz/P4), the RewP from 276 to 376 ms post reward feedback onset over frontocentral channels (FC3/FCz/FC4), and the feedback-P3 from 324 to 424 ms post reward feedback over parietal areas (P3/Pz/P4).

Given that theta activity plays a critical role in effort-based decision-making ([Umemoto et al., 2023](#_ENREF_45)), we extracted theta oscillation in response to choice options in the effort-based decision-making task. The preprocessing stream was identical to the ERP analyses, except that a wider epoch of -1500 to 2000 ms was created. We convoluted single-trial EEG activity (from -1500 to 2000 ms relative to decision option onset) using a complex Morlet wavelet (3–10 cycles, 1–30 Hz, 30 log-spaced frequencies). Following the convolution, epochs were cut from -500 to 1000 ms to account for edge artifacts. Data were normalized by z-scoring a welding baseline (from -500 to -300 ms before choice onset; concatenated across all trials) for each frequency band within participants. Single-trial theta power was measured as mean activity from 100 to 400 ms over 4–7 Hz post decision option onset at FCz, where its activity typically reaches maximum.

**2 Results of behavior data**

*2.1 The effort-reward task*

In the effort-reward task, both groups achieved high success rates (97.77% ± 1.84 for the CNT group and 97.26% ± 2.46 for the ANH group), but their success rates declined as effort levels increased (*b* = -5.73, *p* < .001). Task completion time increased with higher effort (*b* = 1.62, *p* < .001) and decreased with higher rewards (*b* = -0.01, *p* = .005), which was further qualified by a significant interaction between effort and reward (*b* = -0.01, *p* = .031). Follow-up simple slopes analyses at -1 *SD* (“Low”) and +1 *SD* (“High”) effort levels revealed that rewards reduced completion time when required effort was high (*b* = -0.02, *p* < .001) but not when it was low (*b* < -0.01, *p* = .652). Importantly, the ANH group exhibited comparable performance to the CNT group, as demonstrated by similar success rates (*b* = -2.05, *p* = .172) and completion times (*b* = 0.04, *p* = .465). No other significant effects were found (Table 1). Note that the completion time results are based on data from 79 individuals because completion time data of one participant in the ANH group were not recorded.

**Table 1.** Model results of behavior data for the effort-reward (success rates and completion times) and effort-based decision-making (choice rates and times) tasks

|  | Success rates | Completion times | Choice rate | Choice times |
| --- | --- | --- | --- | --- |
| Group | -2.05 | 0.04 | 0.02 | -50.79 |
| Effort | -5.73^***^ | 1.62^***^ | -2.26^***^ | 25.15^***^ |
| Reward | -0.84 | -0.01^**^ | 3.86^***^ | -3.81 |
| Group:Effort | 1.35 | 0.04 | 0.75 | -9.29 |
| Group:Reward | 0.27 | -0.01 | -0.41 | -10.27 |
| Effort:Reward | 0.59 | -0.01^*^ | 0.06 | -3.36 |
| Group:Effort:Reward | -0.22 | -0.00 | -0.11 | 12.98 |

*Note.* Values represent regression coefficients derived from linear mixed-effects models with predictors as noted. Success and choice rates were fitted with a mixed-effects logistic regression model, while completion and choice times were fitted with a linear mixed-effects regression model. ^*^ *p* < .05, ^**^ *p* < .01, ^***^ *p* < .001.

*2.2 The effort-based decision-making task*

Participants’ willingness to invest effort decreased as effort level increased (*b* = -2.26, *p* < .001) but increased with as reward level (*b* = 3.86, *p* < .001). The effort-discounting effect was less pronounced for the ANH group (*b* = -1.88, *p* < .001) than for the CNT group (*b* = -2.64, *p* < .001), as revealed by a marginally significant interaction between group and effort (*b* = 0.75, *p* = .065). Additionally, both groups took longer to make decisions as effort level increased (*b* = 25.15, *p* < .001). No other significant results were found (Tabe 1).

**References**

Delorme, A., & Makeig, S. (2004). EEGLAB: An open source toolbox for analysis of single-trial EEG dynamics including independent component analysis. *Journal of Neuroscience Methods*, *134*(1), 9-21. <https://doi.org/10.1016/j.jneumeth.2003.10.009>

Lopez-Calderon, J., & Luck, S. J. (2014). ERPLAB: An open-source toolbox for the analysis of event-related potentials. *Frontiers in Human Neuroscience*, *8*, 213. <https://doi.org/10.3389/fnhum.2014.00213>

Luck, S. J., & Gaspelin, N. (2017). How to get statistically significant effects in any ERP experiment (and why you shouldn't). *Psychophysiology*, *54*(1), 146-157. <https://doi.org/10.1111/psyp.12639>

**3 Supplementary figure and tables**

**
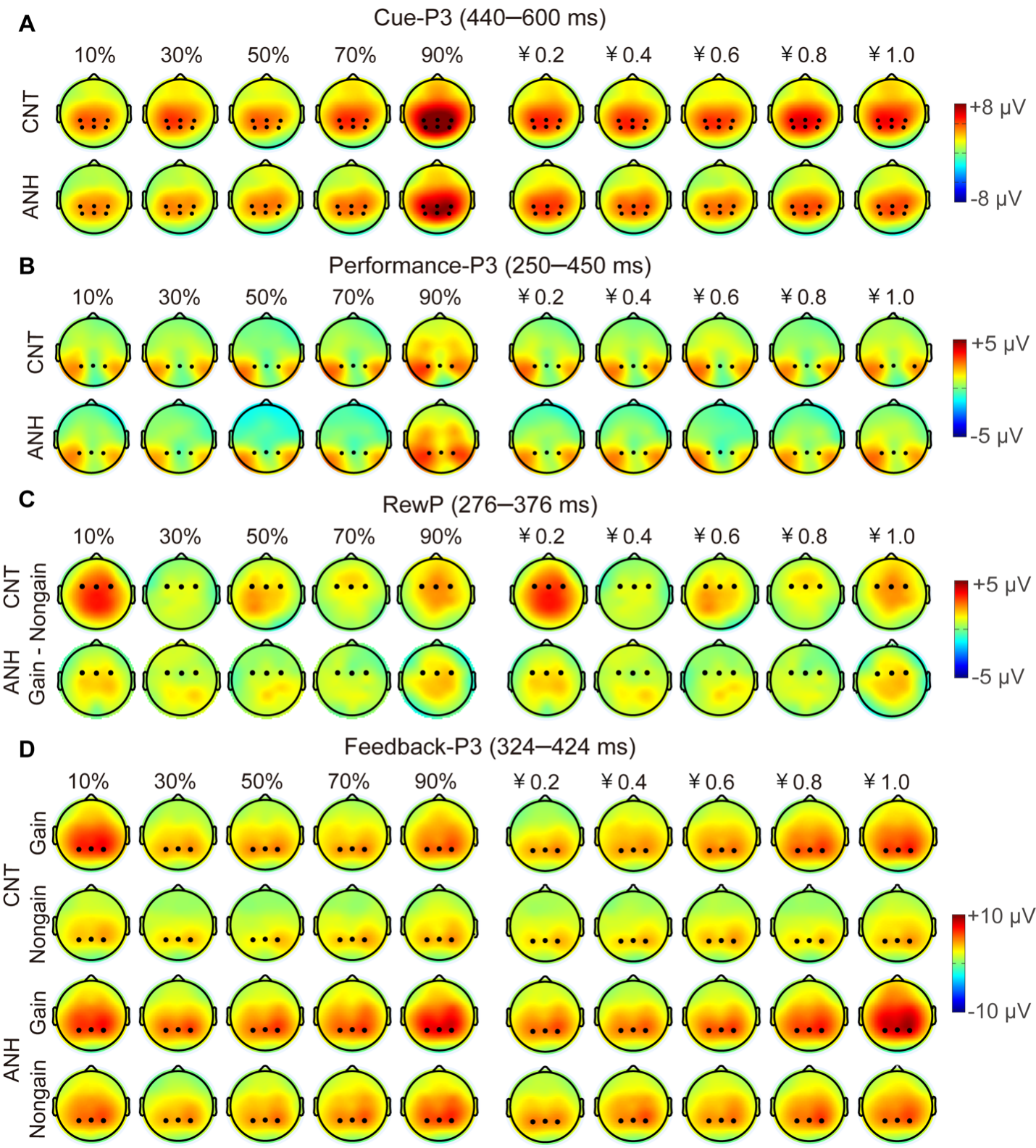
**

**Figure S1.** Topographic maps for the cue-P3 (A), performance-P3 (B), RewP (C), and feedback-P3 (D) as a function of effort level (left) and reward amount (right). The maps for the RewP represent the difference waves between gain and nongain feedback. The black dots represent electrodes used for quantification.

**Table S1.** Model results of rating data

|  | Effort | Liking | Performance | Fatigue | Time pressure |
| --- | --- | --- | --- | --- | --- |
| Group | 0.10 | -0.50 | -0.65^*^ | 0.14 | 0.28 |
| Effort | 2.09^***^ | -1.27^***^ | -1.15^***^ | 2.08^***^ | 2.05^***^ |
| Group:Effort | -0.07 | -0.36^**^ | -0.13 | -0.21 | 0.13 |

*Note.* Values represent regression coefficients of the linear mixed-effects models. ^*^ *p* < .05, ^**^ *p* < .01, ^***^ *p* < .001.

**Table S2.** Model results of EEG data for the effort-reward (the cue-P3, performance-P3, RewP, and feedback-P3) and effort-based decision-making (theta) tasks

|  | Cue-P3 | Performance-P3 | RewP | Feedback-P3 | Theta |
| --- | --- | --- | --- | --- | --- |
| Group | -0.75 | 0.03 | 1.57^*^ | 1.62 | -0.04 |
| Effort | 1.20^***^ | 0.49^***^ | -0.04 | 0.04 | -0.01 |
| Reward | 0.13 | 0.03 | 0.54^***^ | 0.52^***^ | -0.04^***^ |
| Group:Effort | -0.33 | 0.07 | 0.31 | 0.22 | 0.01 |
| Group:Reward | -0.31^*^ | 0.04 | 0.32 | 0.14 | 0.05^*^ |
| Effort:Reward | 0.12 | -0.09 | -0.24^**^ | -0.19^*^ | -0.01 |
| Group:Effort:Reward | -0.03 | 0.12 | 0.22 | -0.11 | 0.06^**^ |
| Valence | – | – | 1.26^***^ | 1.29^***^ | – |
| Group:Valence | – | – | -0.85^*^ | -0.65 | – |
| Effort:Valence | – | – | -0.11 | -0.25 | – |
| Reward:Valence | – | – | 0.42^*^ | 0.71^***^ | – |
| Group:Effort:Valence | – | – | 0.18 | 0.70^*^ | – |
| Group:Reward:Valence | – | – | -0.01 | 0.05 | – |
| Effort:Reward:Valence | – | – | 0.09 | 0.42^*^ | – |
| Group:Effort:Reward:Valence | – | – | 0.04 | 0.03 | – |

*Note.* Values represent regression coefficients derived from linear mixed-effects models. ^*^ *p* < .05, ^**^ *p* < .01, ^***^ *p* < .001.
